# CTLA-4 positive breast cancer cells suppress dendritic cells maturation and function

**DOI:** 10.18632/oncotarget.14626

**Published:** 2017-01-13

**Authors:** Xi Chen, Qianqian Shao, Shengnan Hao, Zhonghua Zhao, Yang Wang, Xiaofan Guo, Ying He, Wenjuan Gao, Haiting Mao

**Affiliations:** ^1^ Institute of Basic Medicial Sciences, Qi Lu Hospital, Shandong University, Jinan, Shandong Province, 250012, P.R.China; ^2^ Department of Neurosurgery, Qi Lu Hospital, Shandong University, Jinan, Shandong Province, 250012, P.R.China

**Keywords:** breast cancer cell, CTLA-4, dendritic cell, CD4^+^T cell, CD8^+^T cell

## Abstract

Cytotoxic T lymphocyte-associated antigen 4 (CTLA-4), a potent immunoregulatory molecule, can down-regulate T-cell activation and inhibit anti-tumor immune response. This study showed that LPS-stimulated human dendritic cells (DCs) decreased the expression of HLA-DR, CD83 and costimulatory molecules (CD40, CD80 and CD86) following coculturing with CTLA-4^+^ breast cancer cells. Moreover, the suppressed DCs further inhibited proliferation of allogeneic CD4^+^/CD8^+^ T-cells, differentiation of Th1 and function of cytotoxic lymphocytes (CTLs). However, CTLA-4 blockade in breast cancer cells could recover DC maturation and cytokine production, elevate antigen-presenting function of DCs, reverse Th1/CTLs response and cytokine secretion. Subsequent study demonstrated that the activation of extracellular-signal regulated kinase and signal transducer and activator of transcription 3 of DCs caused by CTLA-4^+^ breast cancer cells were the predominant mechanism of DC suppression. In addition, CTLA-4 blockade treatment also directly inhibited proliferation and induced apoptosis of CTLA-4^+^ breast cancer cells. Collectively, CTLA-4 was expressed and functional on human breast cancer cells through influencing maturation and function of DCs *in vitro*, and CTLA-4 blockage not only recovered the antigen-presenting function of DCs and T-cells activation but also suppressed the biological activity of breast cancer cells themselves. This study highlights the clinical application of CTLA-4 blockade therapy in breast cancer.

## INTRODUCTION

Breast cancer is the predominant type of cancer among women in both developed and developing countries, representing the leading cause of cancer-related female mortality worldwide. Although significant progress has been made in the diagnosis and therapies, clinical outcomes are still depressing [1]. In recent years, accumulating evidence indicates a functional cross-talk between breast cancer and the immune system. Studies have shown that patients of breast cancer have lower absolute numbers of peripheral blood lymphocytes but increased numbers of functionally suppressive CD4^+^CD25^+^ regulatory T cells (Tregs) in the peripheral blood and tumor microenvironment [2, 3]. Dendritic cells (DCs) obtained from peripheral blood and lymph nodes of patients with operable breast cancer are dysfunctional, with decreased expression levels of MHC Class II and CD86, and decreased IL-12 secretion [4]. It is well known that DCs appear susceptible to tumor-mediated immunosuppression [5]. Both circulating and tumor-infiltrating DCs are functionally impaired in tumor-bearing animals and in cancer patients. The ability of DCs to initiate immune response is strictly dependent on the maturation state. Immature DCs (imDCs) that deficient of costimulatory molecules can induce T-cell anergy, promote alloantigen-specific tolerance, and generate Tregs. That is to say, inhibition of DCs maturation and function is the common mode to evade immune surveillance by tumor cells [6]. It has been reported that *ex vivo* Tregs down-modulate B7-molecules (CD80 and CD86) on cocultured DCs in a cell-contact dependent way and the extent of down-modulation is functionally significant because Tregs-conditioned DCs induce poor T-cell proliferation response [7]. Furthermore, the down-modulation is inhibited by blocking cytotoxic T lymphocyte antigen-4 (CTLA-4, also known as CD152) [7].

CTLA-4, one of the most fundamental immunosuppressive molecules, is a potent negative regulator of T cell response. It is normally expressed on the surface of activated T cells and a subset of Tregs [8]. During the early stage of tumorigenesis, CTLA-4 may elevate the T cell activation threshold, thereby attenuating the antitumor response and elevating tumor susceptibility [9]. In breast cancer there is evidence of increased Tregs levels in circulation and tumor microenvironment [2, 3]. Through constitutive expression of CTLA-4 on Tregs, the interaction of the CD28 ligand on T lymphocytes with the CD80/86 receptor on DCs is blocked, resulting in decreasing of DCs activation, inhibition of IL-12 production, T cell cycle arrest and suppression of CD8^+^ cytotoxic T lymphocytes (CTLs) proliferation [10]. Furthermore, CTLA-4 also leads to down-regulation of T-cell response and peripheral tolerance, diminishes the generation of effective antitumor response, and thus brings tumor immune tolerance. In addition, the natural Tregs, which constitutively express CTLA-4, would be expected to more efficiently engage remaining B7-molecules than the responder T cells, therefore promoting suppression rather than T-cell proliferation [7, 11].

In addition to activated T cells and Tregs, recent studies have confirmed that CTLA-4 is also expressed on nonlymphoid cells of different tissues including liver, skeletal muscle, placental fibroblasts, monocytes, leukemia cells and some solid tumor cells [12]. Contardi E et al. found that CTLA-4 expressed on tumor cells was able to bind with recombinant form of the CTLA-4 ligands CD80/CD86 and induced apoptosis associated with sequential activation of both caspase-8 and caspase-3 [13]. Thus, CTLA-4 expressed on tumor cells may be functional. We have previously demonstrated that CTLA-4 is immune dysregulated in breast cancer and there is a significant increase of CTLA-4 expression not only by T cells from breast cancer patients but also by breast cancer cells themselves. Moreover, elevated expression of CTLA-4 in breast cancer tissues was related to obvious axillary lymph nodes metastases and higher clinical stage [12]. In the present study, we hypothesized that CTLA-4 expressed by breast cancer cells (BCCs) might also interfere with the maturation and function of human DCs in tumor milieu as it did on the Tregs. We have further investigated the effect of CTLA-4 antibody on recovering the maturation and functions of DCs as well as the possible signal transduction pathway involved in conditioned DCs maturation. The direct effects of CTLA-4 antibody on the biological behavior of breast cancer cells were also investigated.

## RESULTS

### CTLA-4 expression in BCCs by flow cytometry

**Figure 1:**
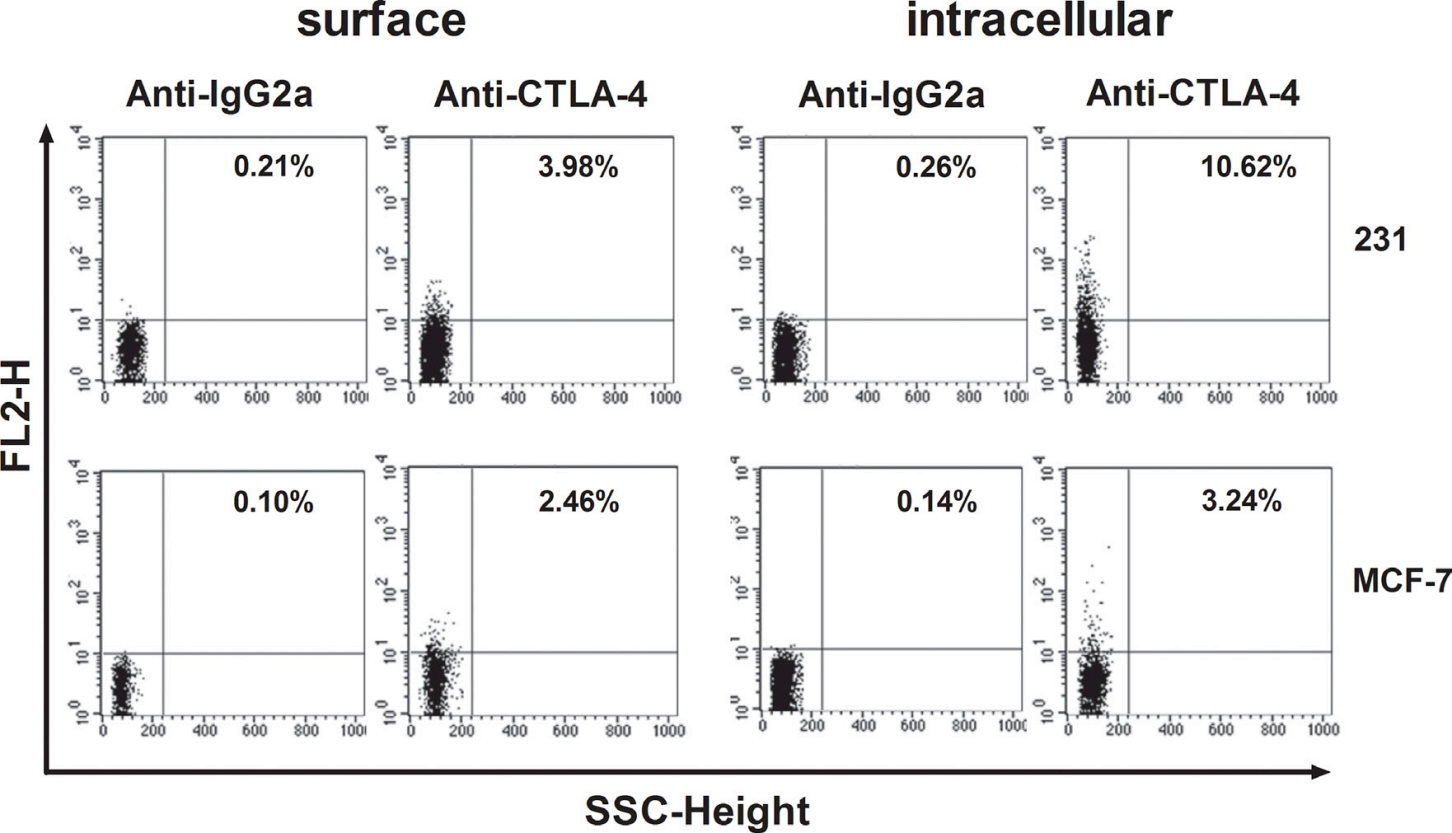
Flow-cytometric analysis of CTLA-4 in BCCs (MDA-MB-231 and MCF-7) MDA-MB-231 and MCF-7 were stained on their surface or intracellularly with the designated antibodies. Results are expressed as percentage of stained cells.

In this study, we first investigated intracellular and surface expression of CTLA-4 in 4 breast cancer cell lines by FACS analysis. As expected, CTLA-4 expression on breast cancer cell lines was detectable, especially MDA-MB-231 (231) and MCF-7 (M7) (Figure 1). Moreover, the intracellular expression was generally higher than the surface expression. The lower levels of surface expression were observed on SKBR3 and T47D (data not shown).

### CTLA-4^+^BCCs inhibit the phenotypic maturation of CD14^+^ monocyte-derived DCs and CTLA-4-blocking could reverse these effects

**Figure 2:**
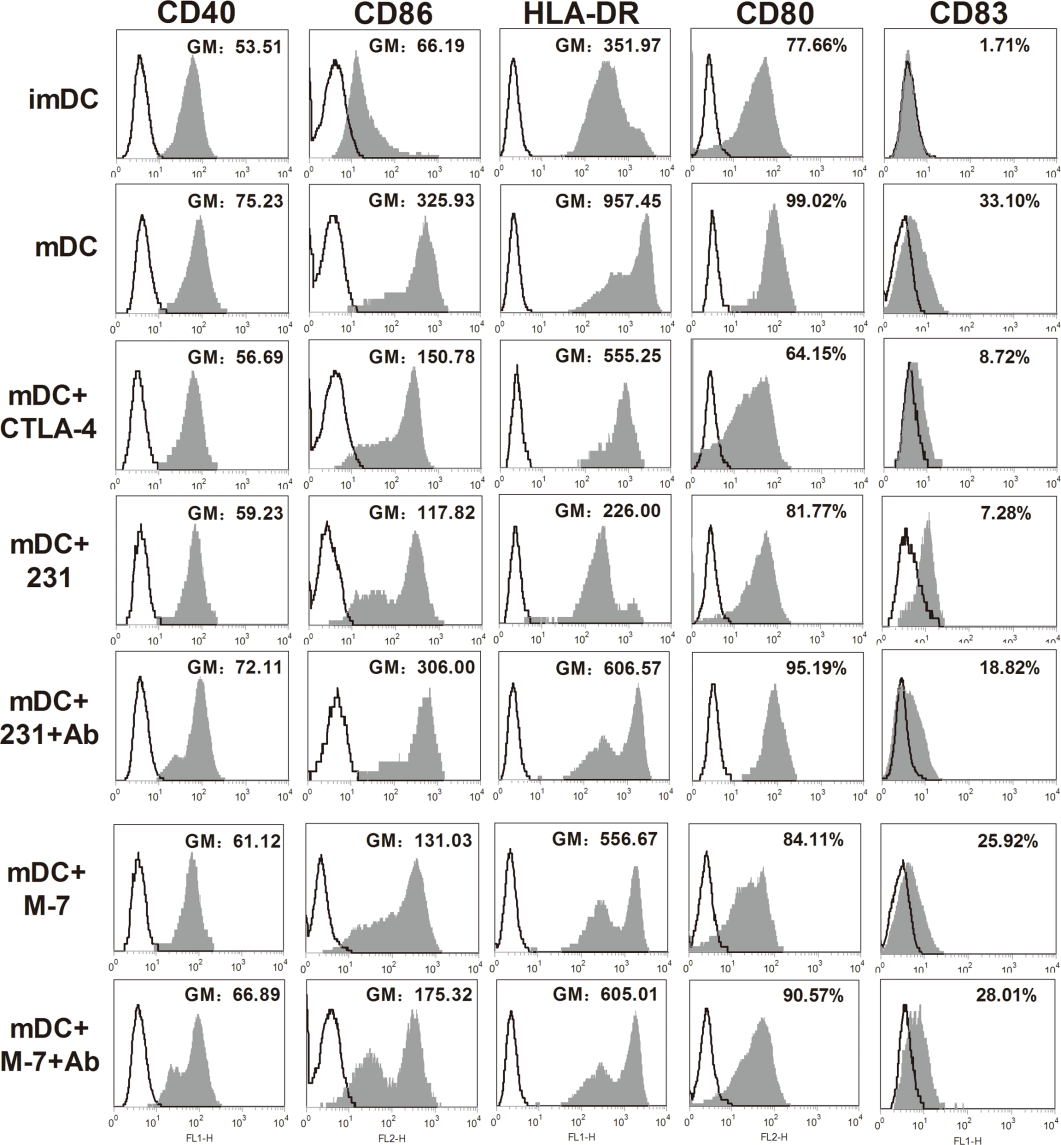
Phenotypic properties of DCs from different treatment groups MDA-MB-231 (231) and MCF-7 (M7) cell lines were used for examining the effects of CTLA-4^+^ BCCs on DCs. Maturation status of DCs was monitored through examining the surface expression of CD40, CD80, CD83, CD86 and HLA-DR. Cancer cells were excluded from analysis by gating on CD11c^+^DCs. DCs were cultured in medium alone, or with soluble CTLA-4-Fc (mDC+CTLA-4), or cocultured with BCCs alone (mDC+231, mDC+M7) or in presence of CTLA-4 mAb (mDC+231+Ab, mDC+M7+Ab), the expression of the surface markers was assayed by flow cytometry. Histograms illustrated staining with isotype-matched control (fine black line) and the positive cells (solid profiles). In these histograms, the expression of CD40, CD86, HLA-DR were presented as geometric mean fluorescence intensities (GM), while the expression of CD80 and CD83 were presented as gated cells (%). Results are representative of at least three independent experiments.

At day 5, human monocyte-derived imDCs were cocultured with CTLA-4^+^BCCs in vitro in the presence of LPS for another 2 days, while soluble CTLA-4-Fc-treated DCs were acted as the positive control. Then the expression of maturation markers on DCs was measured by flow cytometry. As shown in Figure 2, almost all of the surface markers up-regulated in mature DCs (mDCs) were dramatically suppressed in the presence of CTLA-4^+^ BCCs (231 and M7). Consistently, all the suppression effects exerted on CTLA-4^+^BCCs-treated DCs were observed in soluble CTLA-4-Fc-treated DCs. To assess the ability of CTLA-4 expressed on BCCs, we further performed antibody blocking experiment. When a certain concentration of CTLA-4 functional monoclonal antibody (mAb) was added into 231-DC coculture system, the suppressed surface markers were rebounded to different degrees, especially CD40, CD80, CD86 and HLA-DR. However, CTLA-4 mAb did not rebound the suppressed surface markers in M7-DC coculture system. Therefore, BCCs were able to suppress the maturation of DCs by down-regulating the expression of HLA-DR, CD40, CD80, CD83 and CD86, which partially was CTLA-4-dependent. Based on the above results, 231 cell line was chosen as the representative of CTLA-4^+^BCCs for subsequent experiments.

### CTLA-4^+^BCCs inhibit the cytokine production of DCs

**Figure 3:**
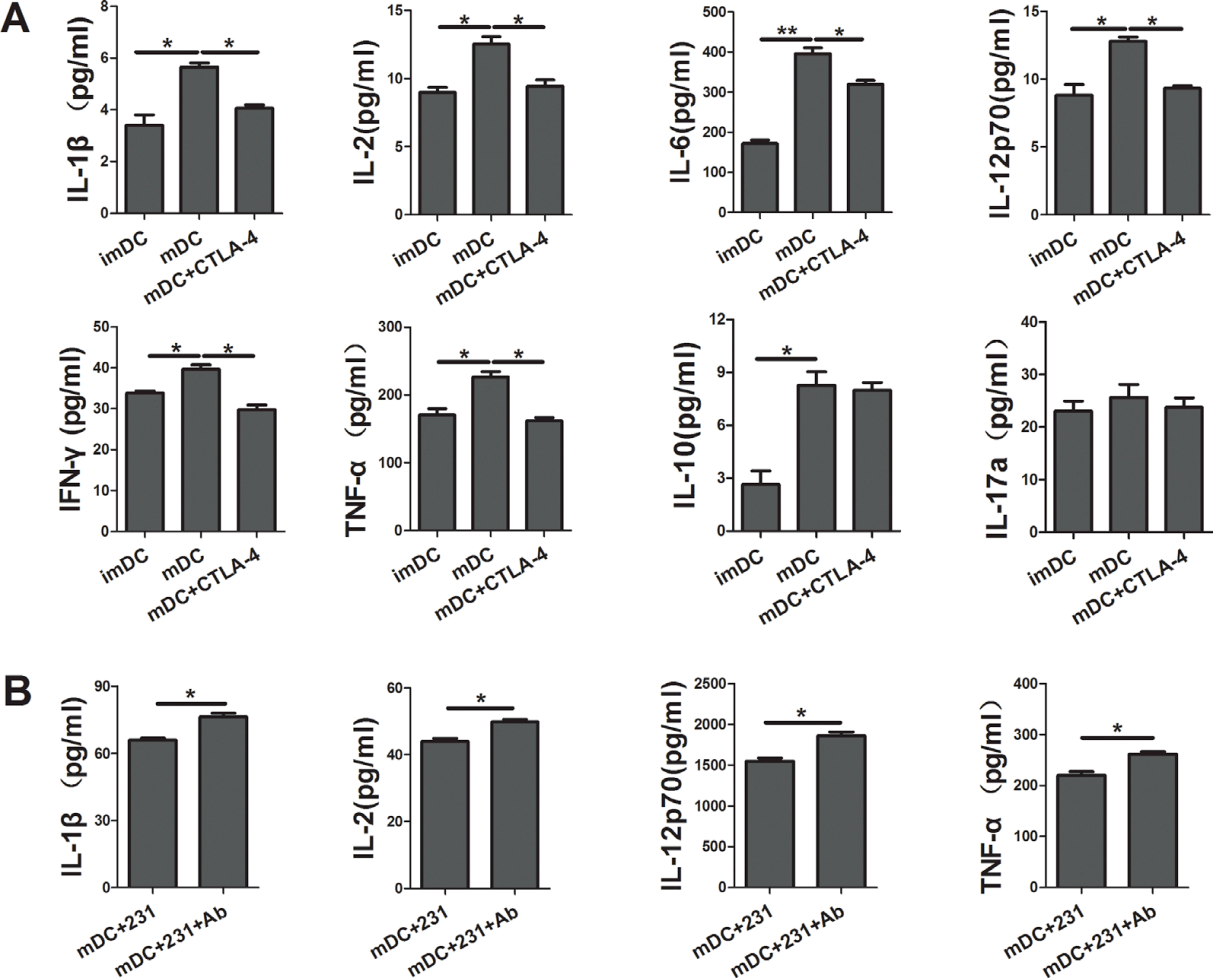
CTLA-4^+^ BCCs inhibit the cytokine production of DCs After coculturing for another two days, the cell-free supernatants of DCs in different coculture were collected and analyzed for the IL-1β, IL-2, IL-6, IL-10, IL-12p70, IL-17a, IFN-γ and TNF-α production by the Bio-Plex Protein Array system. (**A**) The cytokine analysis of imDC, mDC, and mDC+CTLA-4 group. (**B**) The cytokine analysis of mDC+231 and mDC+231+Ab group. Data are presented as mean ± SD from duplicate. *P < 0.05, **P < 0.01.

To further characterize conditioned DCs, we determined the cytokine secretion profile of DCs by Bio-Plex Protein Array system. As shown in Figure 3A, apart from suppressing the maturation of DCs, soluble CTLA-4-Fc could also inhibit the cytokine production of DCs. We found that there was a reduced production of IL-1β, IL-2, IL-6, IL-12p70, IFN-γ, and TNF-α in the supernatant from soluble CTLA-4-Fc-treated DCs, yet the levels of IL-10, IL-17a were not distinctly modulated. Comparing with CTLA-4^+^BCCs-treated DCs, addition of CTLA-4 mAb recovered the cytokine secretion of DCs to some extent, especially IL-1β, IL-2, IL-12p70, and TNF-α (Figure 3B). These data suggested that CTLA-4 blocking was involved in the up-regulation of cytokines production in CTLA-4^+^BCCs treated DCs, which might further recover DCs function in immune response.

### CTLA-4^+^BCCs impair the antigen-presenting cell (APC) ability of mDCs

**Figure 4:**
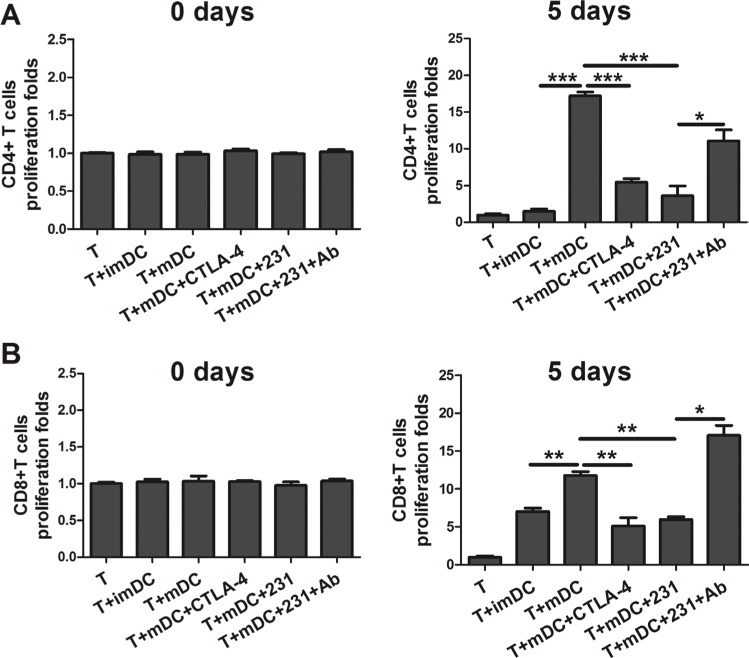
CTLA-4^+^BCCs inhibit the APC function of mDCs Different conditioned DCs (imDC, mDC, mDC+CTLA-4, mDC+231, mDC+231+Ab) were cocultured with allogeneic CD4^+^T cells (**A**) or CD8^+^ T cells (**B**) at ratio of 1:5, the pure T cell proliferation was also monitored as control. The proliferation was measured by CCK8 method on the day 0 and 5, respectively. The bar graphs represent the ratio of CD4^+^ T or CD8^+^ T cells proliferation as mean ± SD of triplicate samples. Results are representative of three independent experiments. *P < 0.05, **P < 0.01, and ***P < 0.001.

Next we asked whether CTLA-4^+^BCCs influenced the APC function of mDCs. To address this question, DCs were cocultured with CTLA-4^+^BCCs, and then tested for their ability to induce the proliferation of allogeneic CD4^+^T cells and CD8^+^ T cells in the mixed lymphocyte reaction (MLR). As shown in Figure 4A–4B, comparing with day 0, all conditioned DCs induced amplification of CD4^+^/CD8^+^ T cells on day 5. As expected, mDCs were more efficient at triggering CD4^+^/CD8^+^ T cells proliferation than imDCs [14, 15]. CTLA-4^+^BCCs-treated and CTLA-4-Fc-treated DCs showed significantly less potency in stimulating both CD4^+^ and CD8^+^ allogeneic T cells proliferation comparing with mDCs. This observation was in keeping with the decreased expression of maturation markers on CTLA-4^+^BCCs-treated DCs. Importantly, CTLA-4-blocking could interfere with suppression of proliferation, because the proliferation of allogeneic CD4^+^/CD8^+^ T cells recovered to almost normal level in coculture. Thus, CTLA-4^+^BCCs inhibited the APC function of mDCs along with its suppression on the phenotypic maturation of mDCs and the inhibitory effect was CTLA-4-dependent.

### CTLA-4^+^BCCs inhibit the ability of DCs to drive the differentiation of naïve CD4^+^ T-cells into Th1 effectors

**Figure 5:**
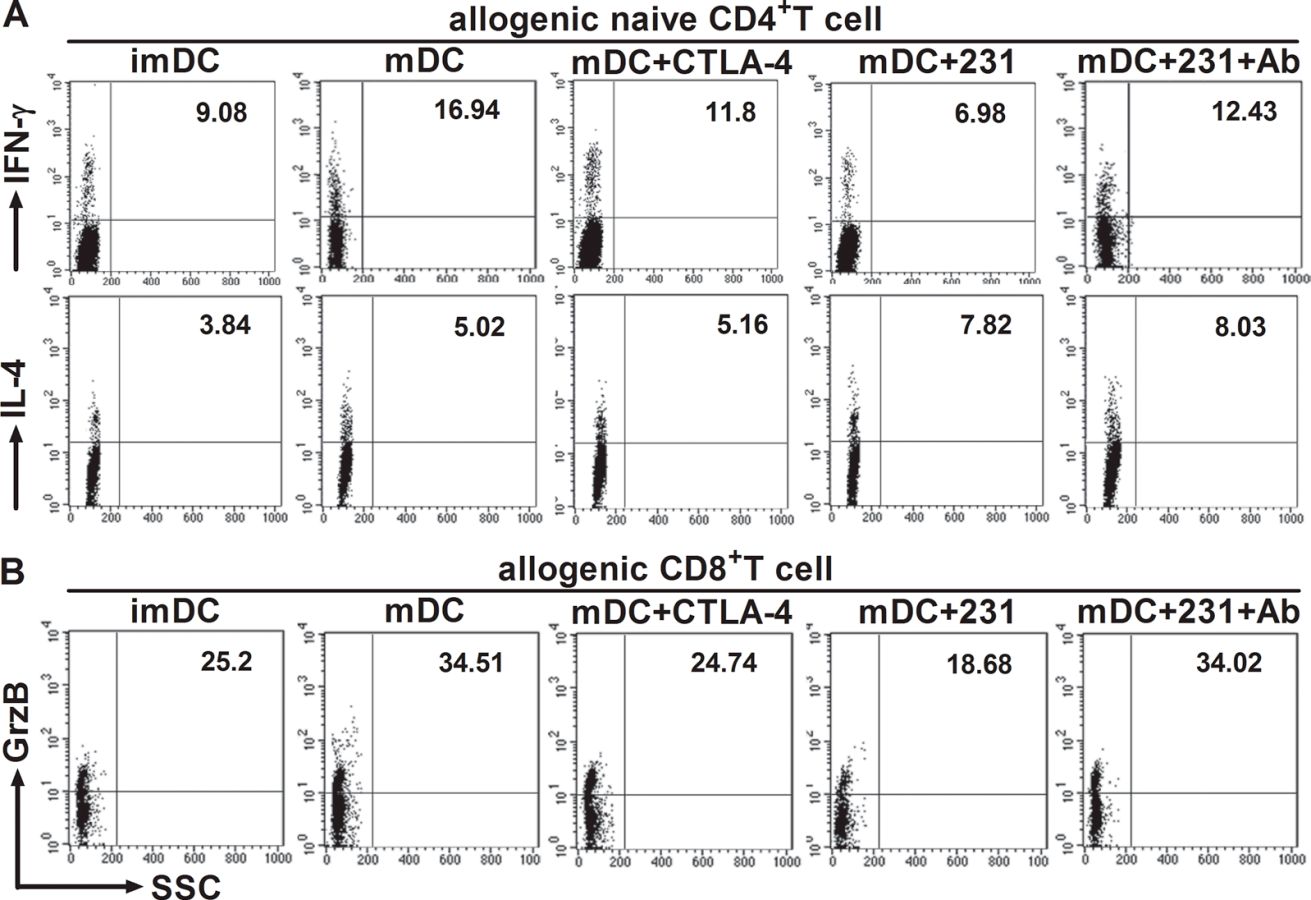
CTLA-4^+^BCCs impair the ability of DCs to induce the response of CD4^+^/CD8^+^ T cell Different conditioned DCs were cocultured with allogeneic CD4^+^ naïve T cells or CD8^+^ T cells to induce T-cell response at a DC: T ratio of 1:5. The percentage of Th1 (IFN-γ^+^), Th2 (IL-4^+^) or CTL (Granzyme B^+^) effectors was assessed by the intracellular staining and flow cytometry on day 5. DCs were excluded from analysis by gating on CD4^+^/CD8^+^ T cells. The numbers in the dot plots indicated the percentage of CD4^+^IFN-γ^+^T cells, CD4^+^IL-4^+^T cells (**A**) or CD8^+^Granzyme B^+^ T cells (**B**). The figure represents one of three similar experiments.

**Figure 6:**
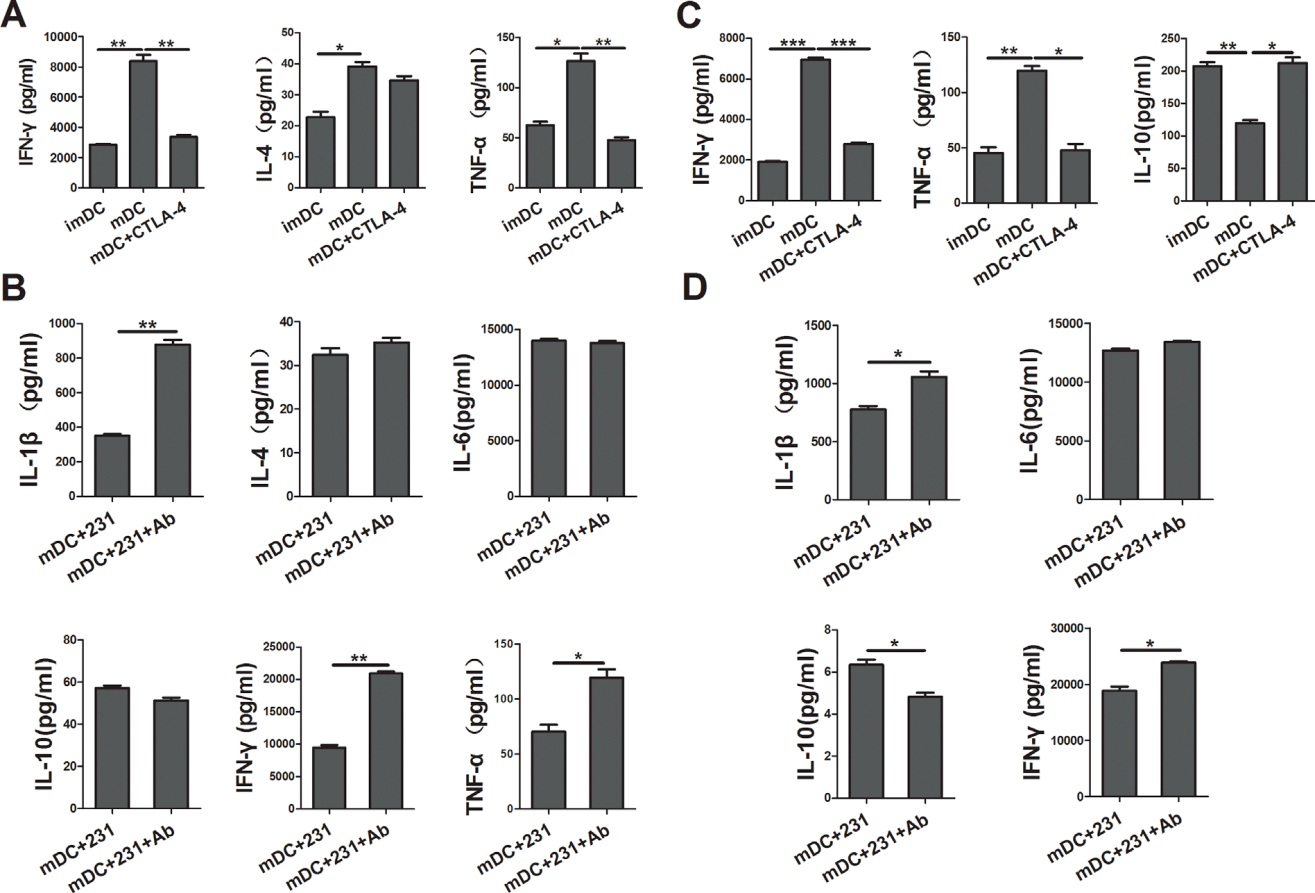
CTLA-4^+^BCCs-treated DCs inhibit the cytokine production in cocultur with CD4^+^/CD8^+^ T cells After different conditioned DCs (imDC, mDC, mDC+CTLA-4, mDC+231, mDC+231+Ab) cocultured with allogeneic CD4^+^ naïve T cells or CD8^+^ T cells for 5 days, the cell-free supernatants were harvested, and the production of IL-1β, IL-4, IL-10, IL-12p70, IL-17a, IFN-γ, and TNF-α was detected by the Bio-Plex Protein Array system. (**A**) and (**B**) represented the cytokine profile (IL-1β, IL-4, IL-6, IL-10, IFN-γ, and TNF-α) in the coculture system of naïve CD4^+^ T cells. (**C**) and (**D**) represented the cytokine (IL-1β, IL-6, IL-10, IFN-γ, and TNF-α) production in the coculture system of CD8^+^ T cells. The data are presented as mean ± SD of duplicate samples. *P < 0.05, **P < 0.01 and ***P < 0.001.

It is well known that the cytokine milieu plays an important role in T-cell differentiation toward either Th1 or Th2 type [16]. Since we observed that CTLA-4^+^ BCCs suppressed the maturation and cytokine production of DCs, we further investigated whether allogeneic naïve CD4^+^ T-cells were responsive to CTLA-4^+^BCCs-treated DCs. To achieve this, allogeneic naïve T cells were stimulated with differently conditioned DCs, and then we monitored the percentage of Th1/Th2 effectors by flow cytometry. A remarkable decrease in the percentage of IFN-γ^+^ Th1 cells was examined in conditioned DCs groups, while there was no distinct change in the percentage of IL-4^+^ Th2 cells (Figure 5A). However, blockade of CTLA-4 rebounded the function of DCs and further recovered the priming of naïve T cells into Th1 effector cells, but no significant changes were observed on IL-4^+^ Th2 cells (Figure 5A). Consistent with the results of intracellular cytokine staining, cytokine secretion profiles showed that CD4^+^ T cells stimulated by CTLA-4-Fc-treated DCs produced less Th1-type cytokine IFN-γ and TNF-α than mDCs did, while the secretion of Th2-type cytokine IL-4 had no significant change (Figure 6A). Similarly, CD4^+^ T cells stimulated by CTLA-4^+^BCCs-treated DCs secreted significantly lower amounts of Th1-type cytokines (IL-1β, IFN-γ and TNF-α) and unchanged Th2-type cytokines (IL-4, IL-6 and IL-10), comparing with CTLA-4 mAb-treated DCs (Figure 6B). Obviously, CTLA-4^+^BCCs indeed inhibited the ability of DCs to induce the differentiation of naïve CD4^+^ T-cells into IFN-γ^+^ Th1 effectors.

### CTLA-4^+^BCCs-treated DCs suppress CD8^+^ T-cells function and antitumor immunity

In the context of cancer, tumor environment evolve to suppress and evade CD8^+^ T-cells [17]. And there was significantly reduced granzyme B production by CD8^+^ T cells from cancer tissue when compared to non-cancer tissue [18]. In the above study, we demonstrated that CTLA-4^+^BCCs impaired APC ability of mDCs and inhibited the differentiation of naïve CD4^+^ T-cells into Th1 effectors. To address the involvement of CTLA-4^+^BCCs-treated DCs in the suppression of CD8^+^ T-cells function, we further monitored the secretion of granzyme B from CD8^+^ effector T cells by intracellular flow cytometry analysis. DCs treated with CTLA-4^+^ BCCs or CTLA-4-Fc showed significantly less potency in stimulating CD8^+^ T-cells to produce granzyme B comparing with mDCs (Figure 5B). While, the percentage of ganzyme B^+^ CTLs was restored in the presence of CTLA-4 mAb (Figure 5B). We then measured the cytokine production by the activated CD8^+^ T-cells through Bio-Plex Protein Array. In accordance with the results of intracellular cytokine staining, CTLA-4-Fc-treated DCs stimulated CD8^+^ T-cells to produce less IFN-γ, TNF-α and higher IL-10 than mDCs did (Figure 6C). And the levels of cytokines IFN-γ, IL-1β, and IL-6 in CTLA-4^+^ BCCs-treated DCs were much lower than those in CTLA-4 mAb-treated DCs, while the secretion of IL-10 was much higher (Figure 6D). Therefore, CTLA-4^+^BCCs might indirectly suppress CD8^+^ T cell function in a CTLA-4-dependent manner.

### CTLA-4^+^BCCs activate ERK and STAT3 during DC maturation

**Figure 7:**
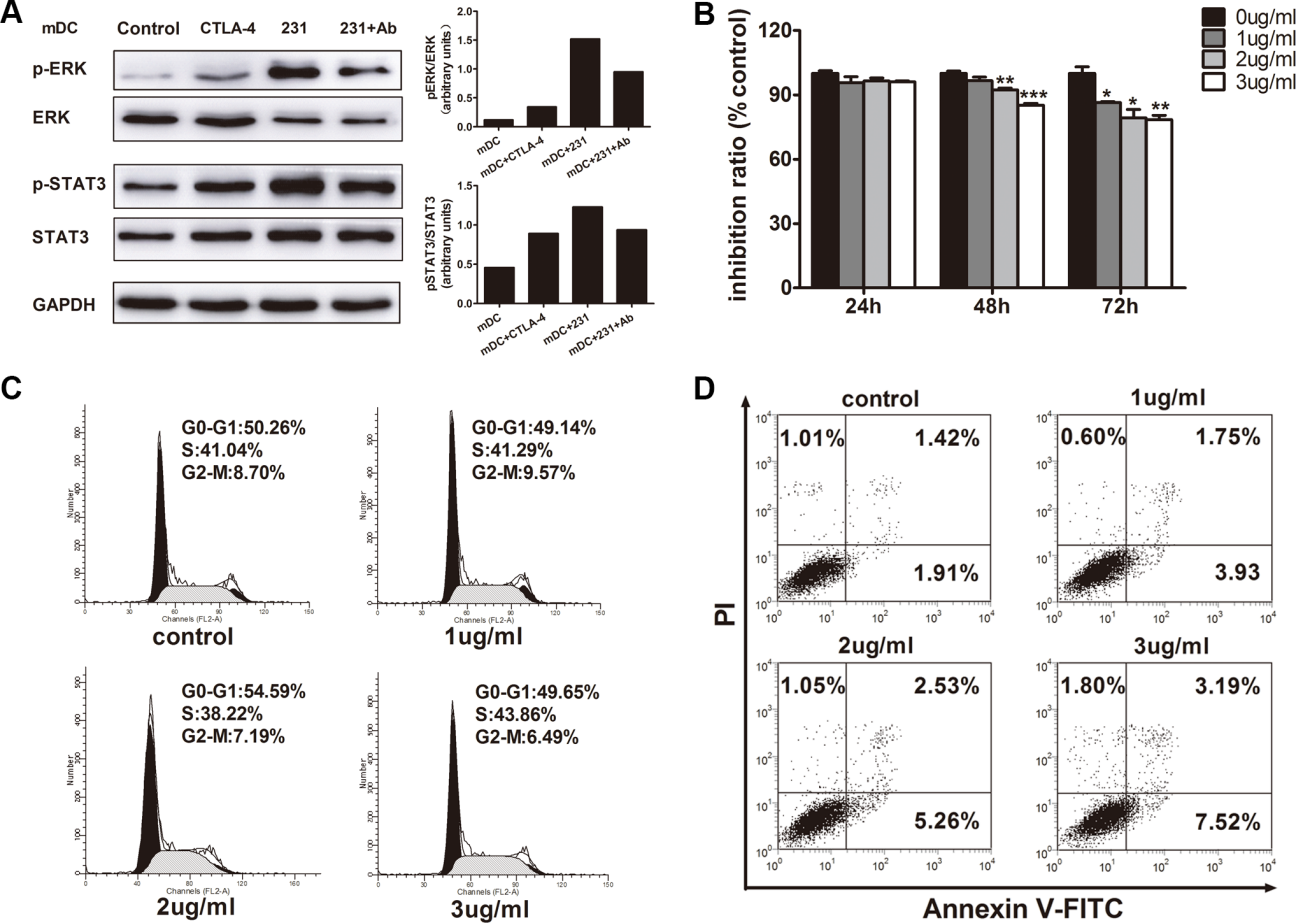
The relevance of ERK and STAT3 activation by CTLA-4 in CTLA-4^+^BCCs-treated DC and the direct inhibitory effects of CTLA-4 mAb on CTLA-4^+^BCCs (**A**) ImDCs were cultured in medium (control), or with soluble CTLA-4 (CTLA-4), or coculture with CTLA-4^+^BCCs alone (231) or in the presence of CTLA-4 mAb (Ab) for 1 hour. Then, treated cells were collected. Whole cell extracts were prepared and the protein levels of phosphorylated (p) and total ERK1/2 and STAT3 were determined by western blot. Right insets: the quantitative analysis of p-ERK/ERK, p-STAT3/STAT3 protein ratio, as measured by ImageJ analysis of band intensity. Values are expressed in arbitrary units. (**B**) CCK-8 assays showed the effects of CTLA-4 mAb on cell viability. (**C**) Flow cytometry assays showed the effects of CTLA-4 mAb on cell cycle progression. (**D**)Annexin staining assays showed the effects of CTLA-4 mAb on cell apoptosis. Representative result from three independent experiments was shown. *P < 0.05, **P < 0.01 and ***P < 0.001.

To understand the molecular mechanisms involved in suppression of DC maturation, we investigated the signaling pathways in CTLA-4^+^BCCs-treated DCs and mDCs. MAPK/ERK pathway was reported to be activated in DCs by LPS and regulated DC survival, while sustained ERK activation in human DCs produced cells with an immature phenotype [19, 20]. STAT3 has been recently characterized as a negative regulator of inflammatory responses and its increased activation in DCs results in impaired T cell response [21]. Abnormal activation of STAT3 was reported to be involved in DC regulatory function under tumor conditioned medium [6]. To detect the ERK and STAT3 induction status in CTLA-4^+^BCCs-treated DCs during DC maturation, we examined the effects on ERK and STAT3 phosphorylation by soluble CTLA-4-Fc, CTLA-4^+^BCCs alone or in combination with CTLA-4 mAb by western blot. As shown in Figure 7A, there was obviously high ERK and STAT3 activation in the DCs treated with soluble CTLA-4-Fc and CTLA-4^+^BCCs compared with that in control mDCs. This was in paralleled with the result that maturation of DCs with LPS in the presence of CTLA-4-Fc resulted in robust phosphorylation of STAT3 and incubation of DCs with tumor lysates increased ERK phosphorylation [21, 22]. In contrast, the ERK and STAT3 activation were remarkably weakened in the CTLA-4 mAb-treated DCs. In addition, level of phosphorylated p38 was also assessed in this study, but we did not find the significant difference among the different groups (data not shown). These indicated that ERK and STAT3 activation may be responsible for CTLA-4^+^BCCs-induced aberration of DC maturation.

### The direct inhibitory effect of CTLA-4 mAb on CTLA-4^+^BCCs

We further explored whether CTLA-4 mAb also had a direct effect on the CTLA-4^+^BCCs as well as DCs. CCK8 assay revealed that CTLA-4 mAb markedly inhibited the growth of CTLA-4^+^BCCs in a dose- and time-dependent manners (Figure 7B). We observed that 3 μg/mL CTLA-4 mAb could significantly reduce cell viability with an incubation period of 72 hours (14.82 ± 2.17%). Based on the above results, we evaluated the influence of CTLA-4 mAb on cell cycle progression. PI staining analysis by flow cytometry demonstrated that CTLA-4 mAb had little influence on cell cycle progression (Figure 7C). Finally, we determined the effects of CTLA-4 mAb on cellular apoptosis. Results from Annexin V-FITC/PI analysis revealed that CTLA-4^+^BCCs treated with CTLA-4 mAb underwent obvious apoptosis than negative cell controls in a dose-dependent manner (Figure 7D). These results suggested that CTLA-4 mAb indeed suppressed the biological activity of CTLA-4^+^BCCs themselves directly.

## DISCUSSION

During tumor development, a complicated and mutual interaction occurs between cancer cells and immune cells [15]. Patients with various malignancies, including breast cancer, show abnormalities in DC number and function [23]. CTLA-4, constitutively expressed at a high level on Tregs, is a negative regulator of T-cell activation which specifically binding to CD80/CD86 molecules expressed on DCs interrupts CD28 mediated signaling to T cells [24]. However, the precise function of CTLA-4 expressed on non-T cells is largely undetected. In this study, we demonstrated for the first time that CTLA-4 expressed on breast cancer cells was functional and CTLA-4^+^BCCs played an inhibitory role on the maturation and function of DCs, which was CTLA-4-dependent.

We first examined the expression of CTLA-4 on breast cancer cell lines and screened out breast cancer cell line MDA-MB-231, which highly express CTLA-4 intracellularly and on cell surface. Whereafter we performed CTLA-4^+^BCCs-DC coculture analysis and the detection of monocyte-derived DC-specific maturation markers. CD80 and CD86 down-regulation is suggested to be an important phenomenon used by Tregs to suppress the DCs in vitro in a CTLA-4-dependent way [7]. Our results showed that CD80 and CD86 on DCs were down-regulated in the presence of CTLA-4^+^BCCs which was similar to those in Tregs. In addition, other DC cell surface markers were also down-regulated obviously by CTLA-4^+^BCCs treatment. Moreover, blockade of CTLA-4 resulted in the remarkable up-regulation of all maturation markers on DCs. The fact that CTLA-4^+^BCCs treatment influenced the expression of surface markers during DCs maturation suggested an immune inhibitory mechanism that may be effective in breast cancer. Further studies to determine whether there are regulatory DCs induced by CTLA-4^+^BCCs would be very interesting.

Another major consequence of CTLA-4^+^BCCs inhibiting DC maturation was the change of cytokine production. Here our results implied that suppression of DC maturation was accompanied by a dramatic decrease in the secretion of the pro-inflammatory cytokines IFN-γ, TNF-α, IL-1β, IL-2, IL-6 and IL-12. Increasing secretion of IFN-γ and IL-12 could promote CD4^+^ T-cells to differentiate into Th1 cells and suppress Th2 formation [25, 26]. On the other hand, TNF-α and IL-6 also activate some other immune cell types, such as macrophages and DCs, in addition to T cells [27, 28]. Therefore, significant decrease in secretion of these cytokines could lead to the inhibition of DC activation by soluble CTLA-4-Fc. However, up-regulation of IL-17a and IL-10 was undetectable in the present study. Furthermore, comparing with CTLA-4^+^BCCs-treated DCs, CTLA-4 mAb-treated DCs showed higher cytokine concentrations of pro-inflammatory cytokines IL-1β, IL-2, IL-12, and TNF-α, which further clearly pointed toward the role of CTLA-4^+^BCCs in inhibiting DC maturation in a CTLA-4-dependent way.

It has been reported that the mechanism to induce tolerance might through abortive proliferation or anergy of antigen-specific CD4^+^ and CD8^+^ T-cells in vivo or through the generation of Tregs to prevent the immune responses by producing IL-10 [29–31]. As expected, in this study, CD4^+^ and CD8^+^ T cells allostimulatory function of mDCs was decreased after treatment with CTLA-4^+^BCCs. Importantly, addition of CTLA-4 mAb restored the proliferation. These data supported the hypothesis that CTLA-4-dependent down-regulation of DC costimulation and cytokine production was a major mechanism used by CTLA-4^+^BCCs to inhibit the peripheral T cell proliferation. As both CD4^+^ and CD8^+^ T cells have been reported to play an important role in immune surveillance, disturbance with the activation of either CD4^+^ or CD8^+^ T cells was likely the critical approach for tumor cells to evade the antitumor immunity from the host [6].

One of the most important findings in the present study was that CTLA-4^+^ BCCs-treated DCs suppressed differentiation of Th1 and function of CTLs. DCs-derived factors mainly balanced the development of these two Th cell phenotypes: Th1 cells mainly secret large amounts of IFN-γ but little IL-4 and IL-10, conversely, Th2 cells can produce large amounts of IL-4 and IL-10 but little IFN-γ [16]. Meanwhile, DC-derived IL-12 is an important cytokine that stimulates IFN-γ production in naïve Th cells and strongly promotes Th1 response [32]. Indeed, we found that both CTLA-4-Fc and CTLA-4^+^BCCs down-regulated IL-12 production of DCs and inhibited the ability of DCs to differentiate naïve CD4^+^ T-cells into IFN-γ^+^ Th1 effectors but not IL-4^+^ Th2 cells. All these further led to the decrease of Th1-type cytokine secretion. In contrast to the results, CTLA-4 mAb treatment rebounded IL-12 and TNF-α secretion of DCs and brought about the recovery of Th1 differentiation and improvement of IFN-γ production by Th1 cells. Subsequently, we focused on the change of CD8^+^ T cell function caused by CTLA-4^+^BCCs-treated DCs. It is reported that there is significantly reduced granzyme B production by CD8^+^ T cells from cancer tissue obtained by lung resection surgery when compared to non-cancer tissue [18, 33]. Local treatment of tumor-bearing mice with CTLA-4 functional antibody in a slow-release formulation is extremely effective in activating an endogenous tumor-specific CD8^+^ T cell response, capable of tumor eradication [34]. Our experimental findings supported the notion that CTLA-4^+^BCCs-treated DCs suppressed the function of CTLs and antitumor immune activation, with a significantly lower ability to stimulate ganzyme B-producing cells. Furthermore, CTLA-4 mAb treatment recovered the function of CTLs through producing higher number of ganzyme B^+^ CD8^+^ T cells which secreted higher level of IFN-γ.

Next, we deeply explored the mechanisms involved in the dysfunction of CTLA-4^+^BCCs-treated DCs. Previous studies have confirmed that aberrant activation of MAPK/ERK in DCs plays a critical role in tumor-induced immune suppression [22], and the relative phosphorylation of ERK is now recognized as a critical regulation of IL-12 generation in DCs [35]. On the other hand, activated STAT3 negatively regulated LPS signaling and was associated with defective DC differentiation mediated by tumor cells or tumor-derived factors [21, 36, 37]. Indeed, in our study, ERK and STAT3 signaling were activated in CTLA-4^+^BCCs-treated DCs, suggesting that activation of ERK and STAT3 may be involved in the defective differentiation of DCs. IL-10 is a well-known STAT3 activators, which has been intimately associated with Tregs-DC crosstalk [21]. However, in our experiments, we did not find increased IL-10 production by CTLA-4^+^BCCs-treated DCs, which might suggest a distinct mechanism of STAT3 activation. Soluble CTLA-4-Fc has been shown to suppress CD80 and CD86 gene transcription and protein production by inducing STAT3 phosphorylation in DCs [21]. Keeping in line with these observations, we demonstrated for the first time that ERK and STAT3 activation of DCs induced by CTLA-4^+^BCCs were responsible for the inhibition of DC phenotypic differentiation and functional performance. Interestingly, we also observed that the total ERK of DCs in coculture systems was generally lower than the DCs alone, the reasonable explanation was that the breast cancer cells regulated the total ERK transcription, this may need further investigation.

Numbers of clinical trials reported that CTLA-4 mAb treatment in patients with metastatic melanoma and prostate cancer could mediate objective clinical regression and enhanced antitumor response by the improvement of effector T-cell activation of patients [38–40]. A question was what effect CTLA-4 mAb could bring on CTLA-4^+^BCCs. In this regard, we found that CTLA-4 mAb could directly suppress proliferation and induce apoptosis of tumor cells. Thus, the therapeutic action of CTLA-4 mAb in tumor may be reduplicated. The precise mechanism behind the antitumor effects of CTLA-4 mAb, however, needed to be investigated further.

In summary, CTLA-4^+^BCCs-treated DCs were phenotypically abnormal and functionally impaired comparing with normal DCs. They expressed significantly lower levels of maturation makers and were poor at activating alloreactive T cells. And these cells were also less potent at inducing Th1 differentiation and CTLs function, which is CTLA-4-dependent. These abnormalities may be attributed to the aberrant activation of ERK and STAT3. Treatment of CTLA-4 mAb not only effectively recovered the function of DCs, but also inhibited proliferation and promoted apoptosis of BCCs. Our study provided evidence for a novel tumor-evading mechanism in breast cancer, and more importantly, suggested a crucial immunomodulatory target, which may help to provide novel immunotherapeutic strategies for breast cancer patients.

## MATERIALS AND METHODS

### Cytokines and monoclonal antibodies (mAbs)

Recombinant human IL-4, GM-CSF, IL-2 and CTLA-4-Fc Chimera were purchased from R&D systems. Lipopolysaccharide (LPS) was from Escherichia coli (Sigma-Aldrich). Functional grade purified anti-human CTLA-4 mAb was purchased from eBioscience (clone 14D3). FITC-labeled anti-human CD4, CD8a, CD14, CD40, CD83, HLA-DR mAb; PE-labeled anti-human CD80, CD86, CD152 (CTLA-4) mAb; PE-Cy5.5-labeled anti-human CD11c mAb and the related isotype controls antibodies were purchased from BD Biosciences. PE-labeled anti-human Granzyme-B, IL-4, IFN-γ mAb and the related isotype controls antibody were purchased from eBioscience.

### Cell lines and cell culture

The following human breast cancer cell lines were purchased from American Type Culture Collection: MDA-MB-231, SKBR3, MCF-7 and T47D. All cells were maintained in RPMI 1640 (Life Technologies/Invitrogen) supplemented with 10% fetal calf serum (FCS) and 1% penicillin/streptomycin (Life Technologies/Gibco). Cells were cultivated in a humidified incubator at 37°C with 5% CO_2_.

### Generation and culture of human monocyte-derived DCs

Human peripheral blood monocytes (PBMCs) were isolated from leukocyte-enriched buffy coats by density gradient centrifugation using Ficoll-Paque Plus (Sigma-Aldrich). The purity of CD14^+^ monocytes from PBMCs was > 98% by positive selection using anti-CD14-conjugated magnetic microbeads (Miltenyi Biotec). Monocytes were cultured for 5 days at 1 × 10^6^/mL in complete RPMI 1640 medium containing 1000 U/mL GM-CSF and 500 U/mL IL-4 to generate imDCs. To obtain mDCs, 1 μg/mL of LPS was added for another 2 days. Purity and phenotype of DCs were assessed by flow cytometry analysis. The use of PBMCs from healthy donors was approved by the Human Investigation Committee of Qilu Hospital, Shandong University, and informed consent was obtained from each subject.

### Isolation of CD4^+^ or CD8^+^ T cells

Human CD4^+^ naïve, CD4^+^ or CD8^+^ T-cells were isolated from PBMCs by immunomagnetic selection using a naïve CD4^+^, CD4^+^ or CD8^+^ T-cell isolation kit (Miltenyi Biotec). The purity was > 97% as confirmed by flow cytometry.

### Coculture of DCs with breast cancer cells (BCCs) or T cells

To investigate the influence of CTLA-4 expressed on the BCCs on the maturation of DCs, imDCs were incubated with BCCs at a ratio of 1:1 in RPMI 1640 supplemented with 10% FCS and LPS in the presence of CTLA-4 mAb (3 μg/mL) or not at the fifth day. BCCs were seeded and incubated in a 37°C with 5% CO_2_ incubator for 8 hours to allow attachment before the addition of DCs. Recombinant human CTLA-4-Fc Chimera (0.8 μg/mL) was added with LPS into imDCs as positive control. After cocultured for another two days, the culture supernatants were collected for cytokine detection by the Bio-Plex Protein Assay System (Bio-Rad). The harvested DCs were analyzed by flow cytometry.

For MLR, these conditioned DCs were further purified and washed with PBS before incubation with allogeneic CD4^+^/CD8^+^ T cells. T cells were cocultured with DCs at T/DC ratio of 5:1 in RPMI 1640 containing 50 U/mL IL-2. After allogeneic DC-T-cell coculture for 5 days at 37°C, the supernatants were harvested for cytokine assay. The harvested T cells were restimulated with 1× cell stimulation cocktail (eBioscience) for another 15–16 hours. In the proliferation activity analysis of T cells, freshly isolated CD4^+^/CD8^+^ T cells were cultured alone or in the presence of different treated DCs at a ratio of 5:1 (T:DC) and placed into 96-well plates. Proliferation rate was detected on day 0 and day 5 of coculture using Cell Counting Kit-8(CCK8) (Beyotime) method.

### Flow cytometry

For cell-surface marker, cells were harvested and incubated with labeled (FITC, PE, or PE-Cy5.5) monoclonal antibodies or appropriate isotype controls on ice for 30 min. Cells were then washed twice and resuspended in 200 ul of cold PBS containing 5% fetal bovine serum. Stained cells were analyzed for single or double or triple color immunofluorescence with a BD Biosciences FACS Calibur flow cytometer. Data analysis was processed using FCS express V3 (De Novo).

For intracellular staining, cells were fixed and permeabilized using intracellular fixation & permeabilization buffer set (eBioscience) according to the manufacture's instructions. While for intracellular cytokine staining, harvested cells were preincubated with 1× cell stimulation cocktail for 15–16 hours. Cells were harvested and stained with FITC-labeled anti-CD4 or CD8 mAb and then with PE-labeled anti-IL-4, IFN-γ, or Granzyme B mAb. All samples were acquired on a BD Biosciences FACS Calibur flow cytometer. Data analysis was processed using FCS express V3 (De Novo).

### Cytokine measurement

Supernatants from DC-BCCs or DC-T coculture system were harvested at the indicated time-points and stored frozen until analysis. Cytokine production of IL-1β, IL-2, IL-4, IL-6, IL-10, IL-12, IL-17A, TNF-α, and IFN-γ were simultaneously determined by the commercially available Human High Sensitivity Panel (eBioscience). The assay was performed according to the manufacture's instructions and each sample was run in duplicate.

### Western blot analysis

To determine the molecular mechanisms involved in DC maturation that cocultured with CTLA-4^+^BCCs, freshly, conditioned DCs were washed three times with cold PBS and lysed in RIPA buffer (Beyotime). The protein concentration was evaluated using the BCA Protein Assay (Beyotime). Equal amounts of protein were then resolved by 10% SDS-PAGE and transferred to polyvinylidene fluoride (PVDF) membrane (Merck Millipore). After blocking with 5% nonfat dried milk, the membranes were probed with primary antibodies (anti-pSTAT3, anti-STAT3 (Abcam), anti-pERK, anti-ERK or anti-GAPDH (Cell signaling) overnight at 4°C. The membranes were then incubated with secondary antibodies linked to horseradish peroxidase and the signals were detected by enhanced chemiluminescence (Life Technology).

### *In vitro* proliferation assay

The direct effect of CTLA-4 mAb on BCCs growth was measured by CCK8 assay. Briefly, BCCs were seeded in triplicate in 96-well plates at 2 × 10^3^ cells/well and left overnight to adhere. Subsequently, the medium was replaced with 200 μl of fresh medium containing different concentrations of CTLA-4 mAb, followed by incubation at 37°C with 5% CO_2_ for 24, 48 and 72 hours. After treatment, 20 μl of CCK8 solution was added to each well for the last 2 hours. The absorbance value was detected at 450 nm wavelength by a Microplate Reader (Bio-Rad, Hercules, CA, USA). Results were representative of three individual experiments. Inhibition ratio (%) = (1-experimental group A450/control group A450) × 100%.

### Cell cycle and apoptosis assay

Cycle arrest and apoptotic cells were detected by flow cytometry analysis. After treatment with different concentration of CTLA-4 mAb for indicated time, CTLA-4^+^BCCs were collected and washed with PBS for three times. For cell cycle assay, the collected cells were stained with propidium-iodide (PI) mixture (1 mg/mL PI (Sigma-Aldrich) + 500 μg/mL DNAse-free RNAse (Qiagen) in PBS). Cellular apoptosis was detected with Annexin V Apoptosis Detection Kit FITC (eBioscience). Stained cells were assessed by flow cytometry, and the data were analyzed by FCS express V3 (De Novo).

### Statistical analysis

All statistical analyses were conducted using GraphPad Prism software. The differences between different groups were analyzed using two-tailed student's *t*-test and data were presented as the mean ± standard deviation. A *P* value of < 0.05 was considered statistically significant.

## Abbreviations

CTLA-4: cytotoxic T lymphocyte-associated antigen 4
Tregs: regulatory T cells
BCCs: breast cancer cells
DCs: dendritic cells
imDCs: immature dendritic cells
mDCs: mature dendritic cells
CTLs: cytotoxic T lymphocytes
ERK: extracellular-signal regulated kinase
STAT3: signal transducer and activator of transcription 3
mAb: monoclonal antibody
LPS: lipopolysaccharide
FCS: fetal calf serum
MLR: mixed lymphocyte reaction
CCK8: cell counting kit-8
